# Intra-pancreatic tissue-derived mesenchymal stromal cells: a promising therapeutic potential with anti-inflammatory and pro-angiogenic profiles

**DOI:** 10.1186/s13287-019-1435-2

**Published:** 2019-11-15

**Authors:** Bashar Khiatah, Meirigeng Qi, Weiting Du, Kuan T-Chen, Kayleigh M. van Megen, Rachel G. Perez, Jeffrey S. Isenberg, Fouad Kandeel, Bart O. Roep, Hsun Teresa Ku, Ismail H. Al-Abdullah

**Affiliations:** 1 Department of Translational Research and Cellular Therapeutics, Diabetes and Metabolism Research Institute, Beckman Research Institute of City of Hope, 1500 E. Duarte Rd, Duarte, CA 91010 USA; 2 Department of Diabetes Immunology, Diabetes and Metabolism Research Institute, Beckman Research Institute of City of Hope, Duarte, CA USA

**Keywords:** Mesenchymal stromal cells, Anti-inflammatory, Angiogenesis, Type 1 diabetes, TSG-6, NRF2, VEGF

## Abstract

**Background:**

Human pancreata contain many types of cells, such as endocrine islets, acinar, ductal, fat, and mesenchymal stromal cells (MSCs). MSCs are important and shown to have a promising therapeutic potential to treat various disease conditions.

**Methods:**

We investigated intra-pancreatic tissue-derived (IPTD) MSCs isolated from tissue fractions that are routinely discarded during pancreatic islet isolation of human cadaveric donors. Furthermore, whether pro-angiogenic and anti-inflammatory properties of these cells could be enhanced was investigated.

**Results:**

IPTD-MSCs were expanded in GMP-compatible CMRL-1066 medium supplemented with 5% human platelet lysate (hPL). IPTD-MSCs were found to be highly pure, with > 95% positive for CD90, CD105, and CD73, and negative for CD45, CD34, CD14, and HLA-DR. Immunofluorescence staining of pancreas tissue demonstrated the presence of CD105^+^ cells in the vicinity of islets. IPTD-MSCs were capable of differentiation into adipocytes, chondrocytes, and osteoblasts in vitro, underscoring their multipotent features. When these cells were cultured in the presence of a low dose of TNF-α, gene expression of tumor necrosis factor alpha-stimulated gene-6 (*TSG-6*) was significantly increased, compared to control. In contrast, treating cells with dimethyloxallyl glycine (DMOG) (a prolyl 4-hydroxylase inhibitor) enhanced mRNA levels of nuclear factor erythroid 2-related factor 2 (*NRF2*) and vascular endothelial growth factor (*VEGF*). Interestingly, a combination of TNF-α and DMOG stimulated the optimal expression of all three genes in IPTD-MSCs. Conditioned medium of IPTD-MSCs treated with a combination of DMOG and TNF-α contained higher levels of pro-angiogenic (VEGF, IL-6, and IL-8) compared to controls, promoting angiogenesis of human endothelial cells in vitro. In contrast, levels of MCP-1, a pro-inflammatory cytokine, were reduced in the conditioned medium of IPTD-MSCs treated with a combination of DMOG and TNF-α.

**Conclusions:**

The results demonstrate that IPTD-MSCs reside within the pancreas and can be separated as part of a standard islet-isolation protocol. These IPTD-MSCs can be expanded and potentiated ex vivo to enhance their anti-inflammatory and pro-angiogenic profiles. The fact that IPTD-MSCs are generated in a GMP-compatible procedure implicates a direct clinical application.

## Background

Mesenchymal stromal cells (MSCs) have the potential for treating various diseases [1]. Currently, over 800 clinical trials involving MSCs have been registered (clinicaltrials.gov), the majority of which are focusing on the application of MSCs to diseases of the musculoskeletal and cardiovascular systems as well as autoimmune type 1 diabetes (T1D) [2, 3]. With respect to the treatment of diabetes with MSCs, some encouraging progress has been made. For example, intravenous injection of umbilical blood-derived allogeneic MSCs improved the function of pancreatic β-cells, reduced the incidence of diabetic complications, and led to insulin independence in some type 2 diabetic patients [4, 5]. Autologous MSCs were used to treat individuals with T1D and lead to the preservation of C-peptide [6]. For this, bone marrow-derived MSCs were aspirated from iliac crest, a procedure with substantial discomfort [6]. Moreover, the administration of bone marrow-derived allogeneic MSCs together with pancreatic islets enhanced islet survival in diabetic non-human primates [7]. These studies employed fetal bovine serum in the MSC culture media, which is less desirable than media that lack animal proteins, pointing to a need for alternative culture and expansion strategies.

The mechanism by which MSCs protect human islets includes the expression of anti-inflammatory and pro-angiogenic genes [8, 9]. Tumor necrosis factor alpha-stimulated gene-6 (*TSG-6*) induced by TNF-α has anti-inflammatory properties [10–12]. Nuclear factor erythroid 2-related factor 2 (*NRF2*) is important in enhancing islet graft survival and function [13, 14]. Additionally, dimethyloxallyl glycine (DMOG), which targets prolyl-4-hydroxylase to prevent the degradation of hypoxia-inducible factor-1α [15] and upregulate vascular endothelial growth factor (VEGF) [16], could be a possible conditioning factor for improving MSC function.

MSCs have been isolated from various sites including subcutaneous adipose tissue [17, 18], bone marrow [19, 20], skeletal muscle [21], umbilical cord blood [22], ocular limbus [23], and amniotic fluid [24]. Blood- and adipose-derived MSCs are widely investigated due to their accessibility, expandability, differentiability, and clinical applicability [25, 26]. During the enzymatic digestion of the cadaveric pancreas, cells are liberated, together with islets, which can then be separated and characterized. In this study, we isolated MSCs from the otherwise discarded fractions of pancreatic tissue. These cells, designated as intra-pancreatic tissue-derived (IPTD) MSCs, were cultured in a GMP-grade and xenoprotein-free culture medium containing human platelet lysate and conditioned in vitro with TNF-α [27] and DMOG. Changes in gene expression, growth factor, and cytokine levels and angiogenic capacity after conditioning were determined. This study identifies a previously unappreciated fraction of the pancreatic digest as a useful source of anti-inflammatory and pro-angiogenic MSCs with possible clinical applications.

## Methods

### Digestion of human pancreata from cadaveric donors

Human cadaveric donor pancreata (*n* = 9) were obtained from an organ procurement organization. Cadaveric donors from which IPTD-MSCs were obtained averaged 33.8 ± 3.1 years of age, 29.8 ± 1.8 body mass index, and 5.1 ± 0.1% hemoglobin A1c (Table 1). Pancreata from individuals with the criteria of Donation after Cardiac Death and HbA1c > 6.5% were excluded from this study. Islet isolation was carried out in a cGMP facility at City of Hope as previously described [28, 29]. Briefly, the pancreas was digested using collagenase supplemented with either thermolysin or neutral protease [28]. The digested pancreatic tissues were collected in 18 250-mL conical tubes and centrifuged at 182×*g*/8 °C for 3 min. Pancreatic tissue was collected, washed, and purified in a cold COBE 2991 cell processor (COBE Laboratories Inc., Lakewood, CA, USA) [30]. Fractions of purified islets were collected and the IPTD-MSCs were cultured as described below.

**Table 1:** Characteristics of donors of pancreata used for islet and IPTD-MSC cell isolation

Isolation number	Age (years)	Race	Sex	HbA1c (%)	BMI	Cause of death
Donor #1	47	C	M	4.7	31.7	CVA
Donor #2	27	H	M	5.2	30.7	T
Donor #3	39	C	M	5	30	ICB
Donor #4	17	H	M	5.2	39.4	HT
Donor #5	38	C	M	5.2	26.3	HT
Donor #6	34	C	M	4.8	33.1	HT
Donor #7	27	H	M	5	24.7	CVA
Donor #8	31	H	M	5.3	31.8	HT
Donor #9	44	AA	M	5.2	20.3	HT
Mean ± SEM	33.8 ± 3.1	NA	NA	5.1 ± 0.1	29.8 ± 1.8	NA

*BMI* body mass index, *HbA1c* hemoglobin A1c, *C* Caucasian, *H* Hispanic, *CVA* cerebrovascular accident, *HT* head trauma, *AA* African-American, *NA* not applicable

### Intra-pancreatic tissue-derived cell harvesting and culture

**Fig. 1:**
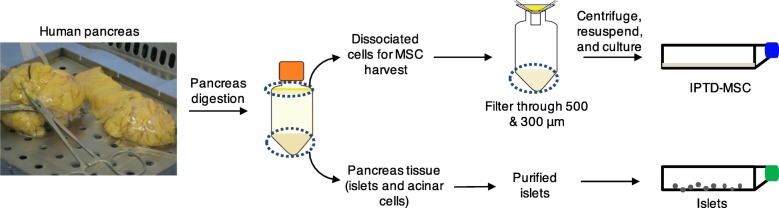
A schematic diagram showing the steps for isolating in a cGMP facility intra-pancreatic tissue-derived cells during human islet isolation

Enzymatic digestion of the whole pancreata released intra-pancreatic tissue and stromal cells. These cells were found to be less dense than the islets and acinar clusters. Under the standard centrifugation condition (182×*g* for 3 min), which was prioritized for islets and acinar clusters, the stromal cells were located at the top layer of the conical tubes (Fig. 1). Until now, this top layer of tissue and cells has been routinely discarded.

To test our hypothesis that IPTD-MSCs can be separated from fractions of the pancreatic tissue, we modified our standard protocol, collected and pooled the upper layer found post-centrifugation, and passed the resultant through double layers of mesh filters (500 and 300 μm) to eliminate non-cellular components (Fig. 1). The filtered cells were then washed with CMRL-1066 culture medium and centrifuged at 727×*g*/8 °C for 3 min. The supernatant was aspirated, and the pellet was suspended in CMRL-1066 culture medium supplemented with 5% Human Platelet Lysate (hPL, Compass Biomed, MA) followed by transferring to a 50-mL conical tube. The suspended cells were centrifuged at 727×*g*/8 °C for 3 min. The supernatant was aspirated, and the pellet was suspended in 40 mL of CMRL-1066 medium containing 5% hPL followed by culturing in T-175 adherent flasks (ThermoFisher Scientific, Waltham, MA) for 24 h at 37 °C in 5% CO_2_ (Fig. 1). Twenty-four hours later, the medium was replaced with fresh CMRL-1066 medium containing 5% hPL. Additional media changes were performed every 48 h until cells reached ~ 80–90% confluence.

### Bone marrow-derived MSCs

Bone marrow-derived human MSCs were obtained from healthy individuals as described [31, 32]. All subjects gave written informed consent in accordance with the Declaration of Helsinki. The study protocol was approved by the Medical Ethics Board of Leiden University Medical Center (LUMC).

### Characterization of IPTD-MSCs

#### Cell morphology

To record the growth and morphology of cultured cells, multiple pictures at different magnifications and time points were obtained using a ckx31 Olympus microscope.

#### Flow cytometry

After reaching 80–90% confluence, cells were dissociated with TrypLE (ThermoFisher, San Diego), washed with DPBS (Corning, Tewksbury, MA) twice, and incubated with antibodies specific for cell-surface molecules, including CD90, CD105, CD73, CD9, CD45, CD34, CD14, and HLA-DR (BioLegends, San Diego, CA), for 20 min at room temperature. In parallel, aliquots of cells were incubated with matched isotype control antibodies from the same supplier. After antibody incubation, cells were washed twice with DPBS and suspended in DPBS for flow cytometry analysis using a Sony SA3800 Spectral Analyzer (Sony Biotechnology, San Jose, CA). Data analysis was performed using Flowjo software (Tree Star, Ashland, OR). To verify the results, human bone marrow-derived MSCs were cultured in the same medium used for IPTD-MSCs, passaged, and expanded in the same procedures for subsequent analysis.

#### Immunofluorescent staining for IPTD-MSCs and pancreatic tissue

Cells were cultured to 70–80% confluence and dissociated into a single-cell suspension using TrypLE as described [33]. Cells and pancreatic tissue were then fixed in 10% cold formalin, prepared in a paraffin block, and sectioned. Antigen retrieval was performed using a citric acid-based antigen unmasking solution (Vector, pH 6.0). Sections were treated with protein block (Biogenex, Fremont, CA) to reduce background signal, followed by incubation with mouse anti-CD105 antibody (ready to use; Biogenex) and ALEXA 488-conjugated goat anti-mouse IgG antibody (1:200 dilution; ThermoFisher). Guinea pig anti-insulin (ThermoFisher) and ALEXA 647-conjugated goat anti-Guinea pig IgG antibodies (1:200 dilution; ThermoFisher) were used for pancreatic tissue staining only. Fluoroshield™ containing DAPI (Sigma Aldrich St. Louis, MO) was used to stain nuclei. Image acquisition was done using an Observer Z1 microscope (Carl Zeiss), with the objective lens set at 20×. Image processing was done using the Zen 2.0 software.

#### Multilineage differentiation of IPTD-MSCs

IPTD-MSCs at the second passage were cultured in T-75 tissue culture flasks until ~ 85% confluence. For adipogenic differentiation, IPTD-MSCs were seeded into 6-well plates and cultured in MesenCult™ Adipogenic Differentiation Kit (STEMCELL Technologies, Vancouver, Canada; Cat# 05412) for 21 days with media changed every 3 days. The presence of lipid droplets in cells was determined by staining with Oil Red O (Sigma, cat# O0625) 21 days after culture. For chondrogenic differentiation, IPTD-MSCs were cultured in two 15-mL conical tubes in MesenCult™-ACF Chondrogenic Differentiation medium (STEMCELL Technologies, Cat# 05455) for 24 days with media changed every 3 days. After culture, Alcian Blue (Sigma, cat#66011) was used to stain for both fresh cells and the cells fixed in paraffin sections. For osteogenic differentiation, cells were cultured in a T-75 tissue culture flask for 22 days with media changed every 3 days. The osteogenic differentiation medium consisted of CMRL-1066 containing 10 mM β-glycerophosphate (Sigma, Cat# G-6251), 50 μg/mL l-ascorbate acid 2-phosphate (Cayman, Item # 16457), 1 μM of dexamethasone (Fresenius Kabi, Cat# 401780G), and 3% hPL. Differentiated cells were fixed in paraffin section and stained with von Kossa for calcium deposition. Undifferentiated IPTD-MSCs were cultured in standard culture medium lacking differentiation factors and stained with Oil Red O, Alcian Blue, or von Kossa.

### In vitro expansion of IPTD-MSCs

T-175 flasks of ~ 80% confluent passage-3 cells were washed twice with DPBS, and 5 ml of TrypLE enzyme was added to each flask. The cells were incubated at 37 °C for 5–10 min to dissociate adherent cells, and 10 ml of CMRL-1066 medium was added to terminate enzyme digestion. Cells were collected in 15-ml tubes for centrifugation at 528×*g* for 3 min. The cell pellet was suspended in 5 ml CMRL-1066 with 5% hPL and vortexed. A sample of cells was mixed in a 1:1 ratio with 0.4% trypan blue (ThermoFisher), from which 20 μL was placed on a counting slide (Cellometer SD100, Nexcelom Bioscience, San Diego, CA) and counted using a Cellometer Auto T4 (Nexcelom Bioscience, San Diego, CA). To further characterize the growth capabilities of these cells, we performed subcultures by placing 5 × 10^4^ cells in T-25 flasks for 72 h at 37 °C and 5% CO_2_. Some cells were grown in CMRL-1066 culture medium alone and others in CMRL-1066 culture medium supplemented with 5% hPL. Culture medium was replaced once during this period. At the end of the culture, cells were dissociated and counted as above. This process was then repeated. After each passage, the cell count was multiplied by the dilution factor to calculate the total number of cells per passage.

### Cryopreservation of IPTD-MSCs

Isolated IPTD-MSCs (at passage 3) were cultured to ~ 80% confluence, dissociated into single cells with TrypLE, collected, and counted. Aliquots of 1 × 10^6^ cells were divided into cryopreservation tubes, suspended in 10% DMSO in CMRL-1066 medium, and stored at − 80 °C in a Mr. Frosty Freezing apparatus containing 100% isopropyl alcohol (ThermoFisher). Using this method, IPTD-MSCs were stored for 9 months. The cells were then thawed rapidly in a 37 °C water bath, washed with DPBS, and cultured in T-75 tissue culture flasks using CMRL media with 5% hPL. After 48 h, the cells were noted to be ~ 80% confluent and were subjected to subsequent analyses. Viability was assessed with trypan blue.

### In vitro treatment of IPTD-MSCs with TNF-α and DMOG

Recombinant human TNF-α protein (R&D Systems, Minneapolis, MN) was reconstituted in research-grade water (Hospira, Lake Forest, IL) to a concentration of 100 ng/mL, aliquoted, and stored at − 20 °C. Dimethyloxallyl glycine (DMOG; Cayman Chemicals, Ann Arbor, MI) was dissolved in water to yield a stock solution of 57.1 mM, aliquoted (100 μL), and stored at − 80 °C. IPTD-MSCs were incubated in T-25 flasks in 5 ml of CMRL-1066 medium supplemented with 5% hPL until ~ 50% confluent. Cells were cultured for 24 h in CMRL 1066 medium, or medium containing 10 ng/ml TNF-α, 1 mM DMOG or 1 mM DMOG and 10 ng/ml TNF-α. Following treatment, cells were collected in 1.7-ml Eppendorf tubes and stored in RLT buffer (Qiagen, Germantown, MD) at − 80 °C for future preparation of cDNA.

### Quantitative real-time PCR

The TaqMan Gene Expression Assay system (ThermoFisher Scientific) was used to quantify *β-ACTIN*, *TSG-6*, *NRF2*, and *VEGF* mRNA levels. Total RNA was extracted using a Qiagen Mini Kit (Cat. No. 51306) and converted into cDNA. Real-time quantitative PCR was run in duplicate on a ViiA™ 7 Real-Time PCR System with a 384-well block (ThermoFisher Scientific). Thermal cycles were programmed for 20 s at 95 °C for the initial denaturation, followed by 45 cycles of 120 s at 95 °C for denaturation, 30 s at 60 °C for annealing, 60 s at 72 °C for extension, and a final extension at 72 °C for 10 min. All PCR runs were performed with negative (water) and positive controls. *β-ACTIN* was used as an internal control to quantify relative gene expression.

### Cytokine assay

Supernatants from cells cultured for 24 h under various conditions (medium alone, or medium plus DMOG, TNF-α, or DMOG + TNF-α) were collected, and cytokine analysis performed using a Luminex assay kit (Bio-Rad, Hercules, CA) according to the manufacturer’s protocol. The following growth factors/cytokines were measured: VEGF, IL-6, IL-8, MMP-9, MCP-1, MMP-2, IL-4, IL-10, and IL-1β. Samples were measured in duplicate.

### In vitro angiogenesis assay

Angiogenic capacity was assessed by quantifying endothelial tube formation [34]. Human umbilical vein endothelial cells (HUVECs) (Cell Applications Inc., San Diego, CA; Cat# 200p-05n) between passages 2 and 6 were cultured in a standard medium. Cells (1 × 10^5^ cells/well) were plated in 24-well plates (Fisher, Cat # 930186) coated with Matrigel (Corning, Cat# 356234), and incubated for 30 min to allow cell attachment. Supernatants (150 μL/well) from control and stimulated IPTD-MSCs (DMOG, TNF-α, or DMOG + TNF-α) were added to obtain a total volume of 300 μL per well. Plates were then incubated at 37 °C, 5% CO_2_ for 24 h. At 4 and 24 h, the wells were visualized using a Leica microscope and representative photographic images were obtained. Total endothelial tube number and tube length were determined using ImageJ software (NIH, Bethesda, MD).

### Statistical analysis

Data was analyzed with GraphPad Prism software (GraphPad Software 8.0, La Jolla, CA). ANOVA one-way analysis of variance was used to compare multiple experimental groups followed by the Tukey multiple comparisons test to compare the mean values between any two groups. All the values were expressed as mean ± standard error of mean (SEM). For all the tests, *p* < 0.05 was considered significant.

## Results

### A chemically defined medium supports the growth IPTD-MSCs

**Fig. 2:**
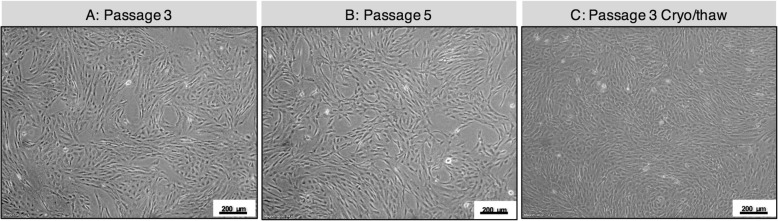
IPTD cells resemble MSCs in culture and can be cryopreserved Phase contrast microscopy of IPTD-MSCs cells cultured in CMRL-1066 medium supplemented with 5% hPL. **a** Passage 3 cell culture on day 3; **b** passage 5 cell culture on day 3; **c** passage 3 cells after 9 months of cryopreservation, thawing, and culture on day 3. Representative images are presented. Scale bar, 200 μm

To develop a GMP-compatible culture medium, we tested whether hPL could support the growth of IPTD-MSCs in the absence of fetal bovine serum. Under phase-contrast microscopic evaluation, IPTD-MSCs displayed elongated and spindle shapes (Fig. 2a), a morphology consistent with the classic MSCs derived from other tissues [35]. Next, we dissociated and replated IPTD-MSCs multiple times and found that the spindle shape morphology was preserved throughout numerous passages (Fig. 2b). IPTD-MSCs were cryopreserved for 9 months, thawed, and cultured, and again the cell morphology remained stable (Fig. 2c).

### hPL is required for the growth of IPTD-MSCs

**Fig. 3:**
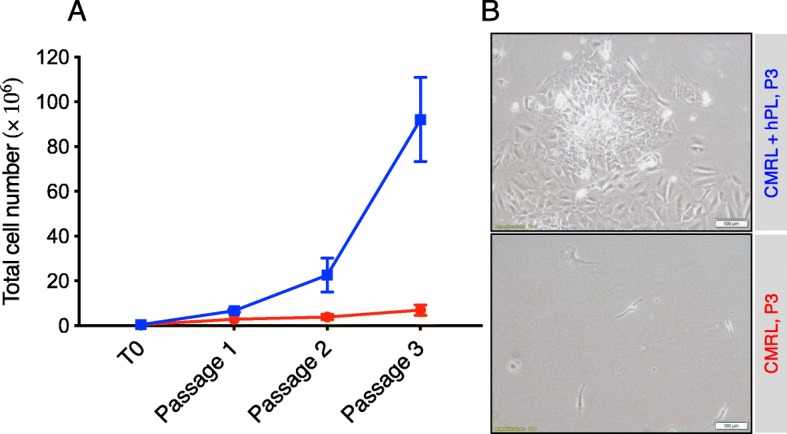
The growth of IPTD-MSCs is enhanced by a culture medium supplemented with hPL. **a** Total cell numbers during expansion under the designated culture conditions. hPL was essential for the expansion of cells in vitro. The results shown were from three different tissue donors; **b** A representative photomicrograph of passage 3 cells cultured in CMRL-1066 culture medium with (green) and without (red) 5% hPL. Scale bar, 100 μm

We tested whether hPL is essential for the growth of IPTD-MSCs by removing it from the culture medium and passaging cells for three generations. IPTD-MSCs grown in hPL-repleted medium expanded an average of 10.8-fold, while those cultured in medium lacking hPL showed minimal to no expansion (Fig. 3), suggesting that hPL is required for the expansion of IPTD-MSCs in vitro.

### IPTD-MSCs display classic MSC cell-surface markers

**Fig. 4:**
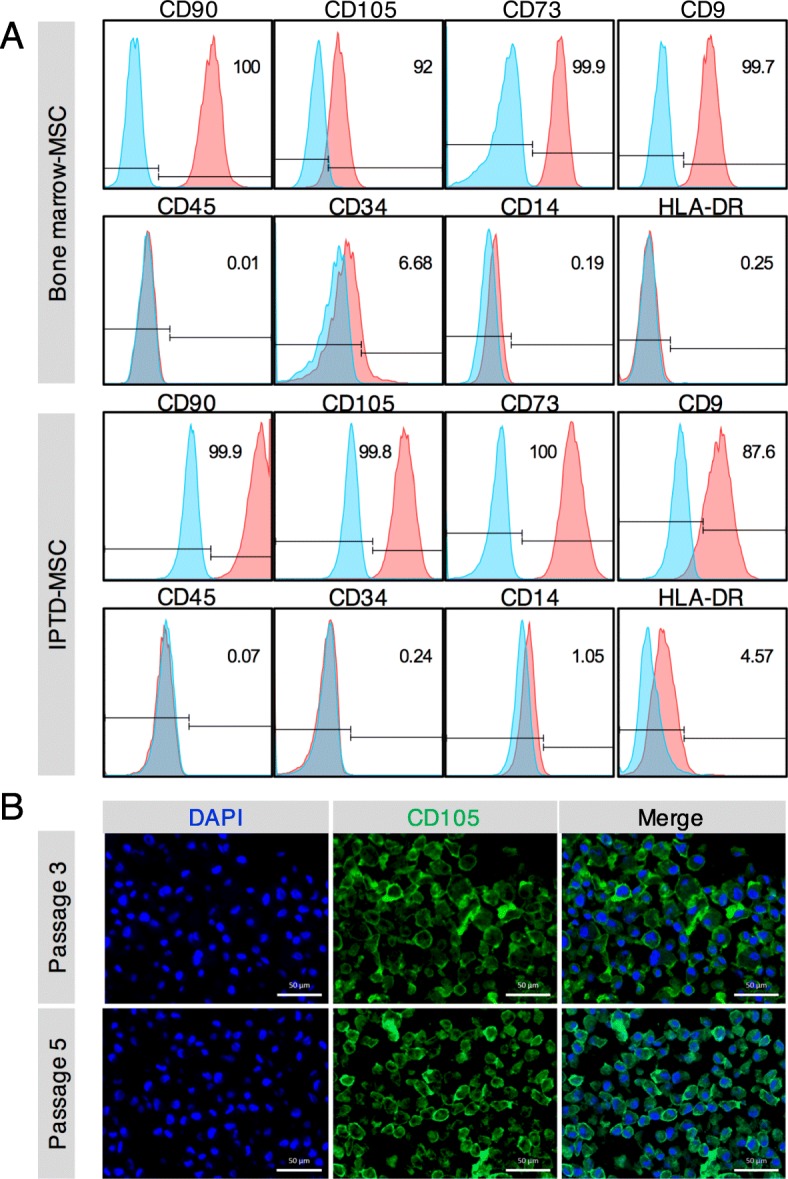
IPTD-MSCs display a cell-surface protein profile consistent with classic MSCs. **a** Flow cytometry analysis of cell-surface protein expression of bone marrow-derived MSCs and IPTD-MSCs. Data are representative of four pancreas donors. **b** Immunofluorescent staining for CD105 protein expression (green) in paraffin sections of IPTD-MSCs (passages 3 and 5) grown in the presence of hPL. Scale bar = 50 μm. DAPI stains nuclei

To test whether IPTD-MSCs express known markers found on MSCs isolated from bone marrow and other organs [36], we performed flow cytometry analysis. We first confirmed cell-surface marker expression using bone marrow-derived MSCs. As expected, the majority of bone marrow-derived MSCs expressed CD90, CD105, CD73, and CD9 and showed minimal to no expression of CD45 (pan-leukocytes), CD34 (hematopoietic cells), CD14 (macrophages), and HLA-DR (antigen-presenting cells) (Fig. 4a).

IPTD-MSCs were passaged 3 times, dissociated into a single-cell suspension and stained with the above mentioned antibodies. Compared to isotype-control staining, the vast majority of IPTD-MSCs stained positive for CD90 (99.2 ± 0.3%), CD105 (99.8 ± 0.2%), CD73 (99.6 ± 0.3%), and CD9 (86.8 ± 2.6%) (Fig. 4a). Minimal expression of CD45 (0.3 ± 0.2%), CD34 (0.3 ± 0.0%), CD14 (1.5 ± 0.8%), and HLA-DR was found (Fig. 4a). Expression of CD105 on the cell surface of IPTD-MSCs at passages 3 and 5 was further visualized using immunofluorescent staining (Fig. 4b). Taken together, IPTD-MSCs expressed classic positive and lacked negative markers for MSCs, suggesting that they reside within the MSC family of cells.

### CD105^+^ cells localize in the pancreas near insulin-expressing cells

**Fig. 5:**
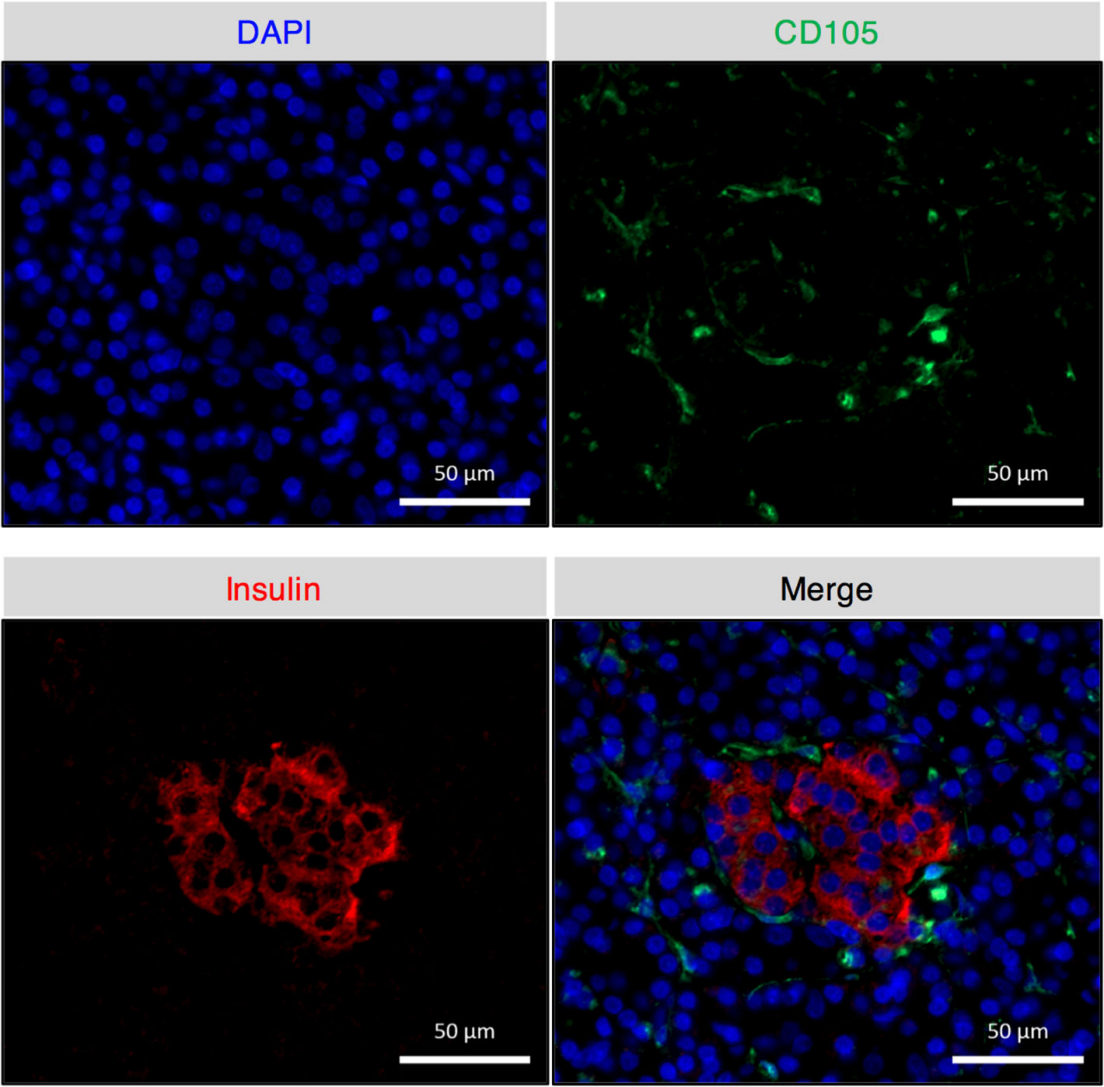
Cells expressing CD105 are found in non-digested pancreatic tissue. Double immunofluorescent staining for CD105 (green) and insulin (red) revealed that CD105-positive cells are present in the pancreatic tissue and located adjacent to the insulin-expressing islets. Photomicrographs were obtained using a Z1 microscope (Carl Zeiss) at flourescence wavelengths of 488 nm (CD 105) and 647 nm (insulin). Images are representative of 3 separate experiments. Scale bars, 50 μm

To rule out the possibility that the ex vivo IPTD-MSC growth and expansion was due to in vitro selection or artifact, we examined whether CD105^+^ cells were present in the endogenous pancreas. Pancreatic tissue sections were co-stained with CD105 and insulin. CD105^+^ cells were detected in the pancreatic tissue and were located adjacent to the insulin-expressing islets (Fig. 5). This result confirms the existence of CD105^+^ cells in the adult human pancreas.

### IPTD-MSCs have potential to differentiate into multiple cell lineages in vitro

**Fig. 6:**
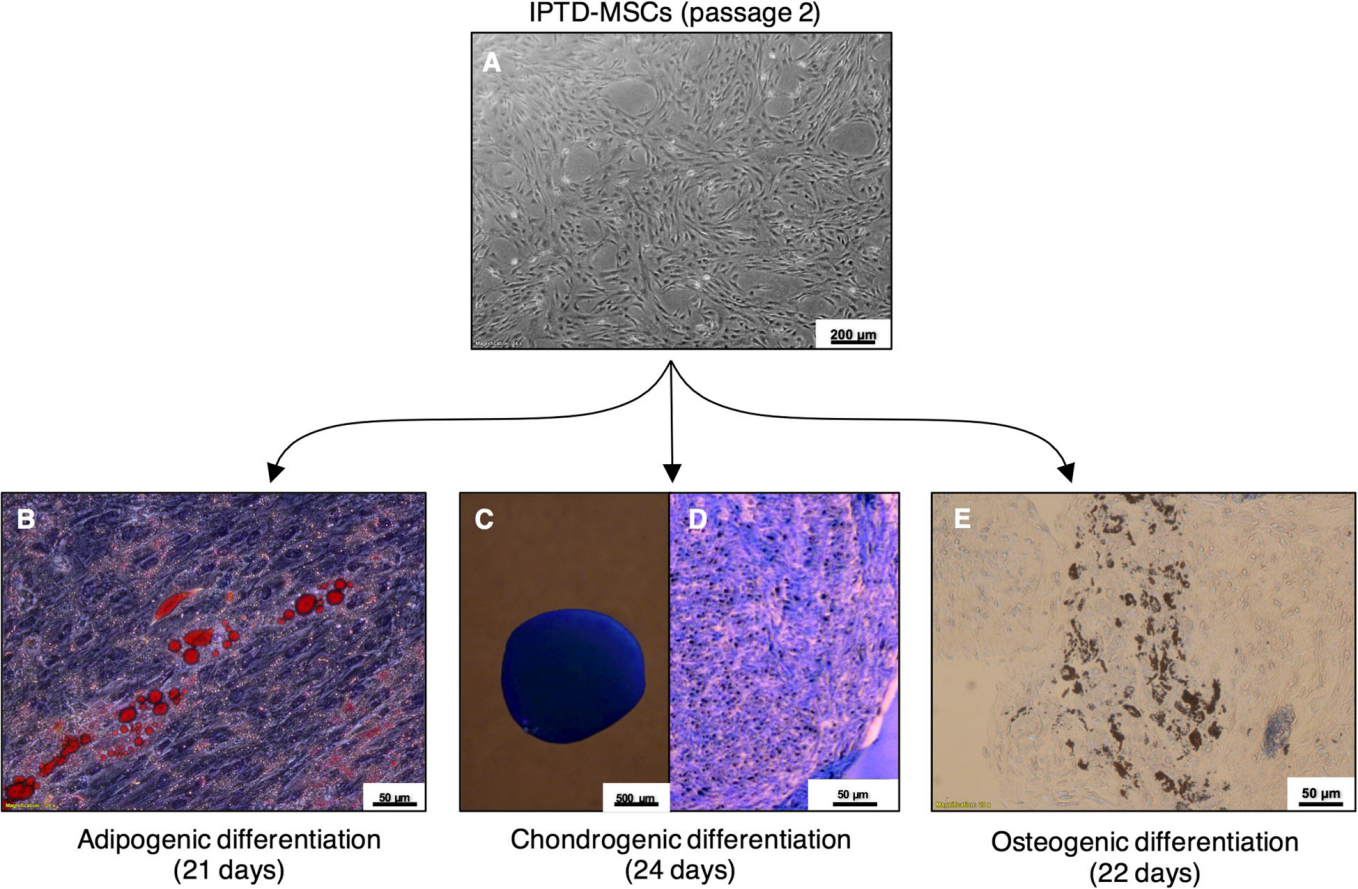
IPTD-MSCs can be differentiated into adipogenic, chondrogenic, and osteogenic cells. (**a**) Passage 3 of IPTD-MSCs prior to differentiation. (**b**) Lipid droplets are detected after staining with Oil Red O indicating that IPTD-MSCs are undergoing adipogenic differentiation 21 days post culture. (**c**) The presence of cartilage is confirmed after staining with Alcian Blue showing in dark blue color. (**d**) Calcium deposition is detected in paraffin-fixed tissue section of the cells cultured in chondrogenic media after staining with Alcian Blue. (**e**) Calcium deposition is detected in paraffin-fixed tissue section of the cells cultured in osteogenic medium after staining with von Kossa

A typical feature of MSCs is the ability to assume lineage-specific cell phenotypes after exposure to certain growth factors. IPTD-MSCs were exposed to adipogenic, chondrogenic, and osteogenic growth conditions. Under these differentiation conditions, IPTD-MSCs were found to give rise to the appropriate lineage-associated phenotypes, including cells positively stained for Oil Red O (adipocytes), Alcian Blue (chondrocytes), or von Kossa (osteoblasts) (Fig. 6). In contrast, undifferentiated IPTD-MSCs showed no lineage-specific staining.

### TNF-α and DMOG upregulate immune-regulatory and angiogenic genes in IPTD-MSCs

**Fig. 7:**
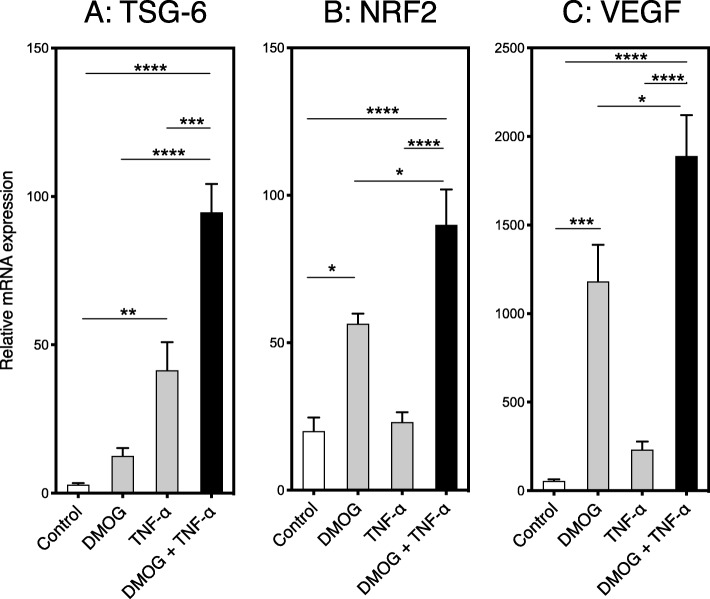
IPTD-MSCs treated with TNF-α and DMOG display increased mRNA levels of anti-inflammatory and pro-angiogenic genes. **a** *TSG-6*, **b** *NRF2*, and **c** *VEGF* mRNA levels from IPTD-MSCs treated with TNF-α (10 ng/mL) and/or DMOG (1 mM). Three independent donors were tested. The data are expressed as mean ± SEM. *p < 0.05; **p < 0.01; ***p < 0.001; ****p < 0.0001

Next, we tested whether IPTD-MSCs were amenable to in vitro conditioning. IPTD-MSCs were stimulated with TNF-α and/or DMOG, and the expression of known immune-modulating and angiogenic genes was determined. Consistent to previous findings [27], *TSG-6* gene expression levels were significantly increased in cells treated with 10 ng/ml TNF-α (*p* < 0.01) but not DMOG alone (Fig. 7a), compared to control. Addition of DMOG to TNF-α further increased the expression of TSG-6 (*p* < 0.0001) (Fig. 7a). *NRF2* and *VEGF* expression were significantly increased when cells were treated with DMOG (*p* < 0.05 and *p* < 0.001 respectively, Fig. 7b, c) but not TNF-α alone, compared to control. Addition of DMOG to TNF-α further enhanced the expression of *NRF2* and *VEGF* (*p* < 0.0001, Fig. 7b, c). These results suggest that, while TNF-α and DMOG display divergent effects, the combination of the two best enhances in IPTD-MSC genes that are known to modulate immune responses and angiogenesis.

### TNF-α and DMOG alter growth factors and cytokines released by IPTD-MSCs

**Fig. 8:**
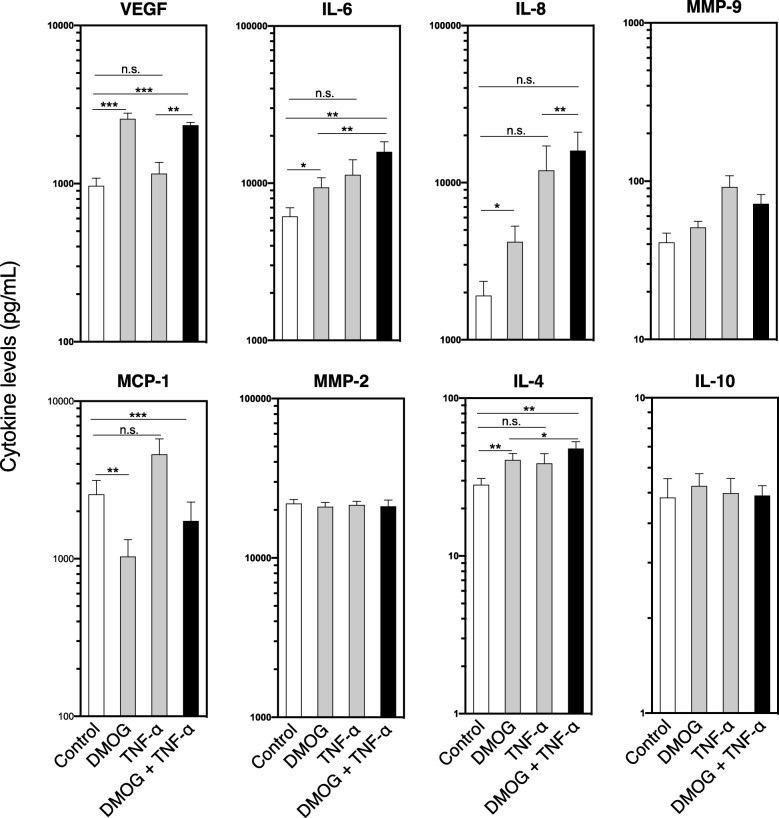
IPTD-MSCs treated with TNF-α and DMOG produce increased levels of several cytokines. Relative changes of the indicated cytokines and growth factors found in medium from conditioned and control IPTD-MSCs. *Y*-axis is logarithmic. Four independent experiments were performed. The data are expressed as mean ± SEM. *p < 0.05; **p < 0.01; ***p < 0.001; ****p < 0.0001; n.s. not significant

To further characterize IPTD-MSCs, we examined proteins released from these cells. IPTD-MSCs were cultured for 24 h in the presence of exogenous TNF-α, DMOG, or both, and the resulting culture media were examined by Luminex assay. Compared to control, stimulation of IPTD-MSCs with DMOG alone enhanced the secretion of VEGF, IL-6, IL-8, and IL-4 (Fig. 8; comparing the 1st to the 2nd bars). Stimulation of IPTD-MSCs with the combination of DMOG plus TNF-α enhanced the secretion of VEGF, IL-6, and IL-4 (Fig. 8; comparing the 1st to the 4th bars), while the stimulation of IPTD-MSCs with TNF-α alone did not have an effect on any of the cytokines examined compared to controls. The addition of TNF-α to DMOG enhanced the secretion of IL-6 and IL-4 (Fig. 8; comparing the 2nd to the 4th bars). Levels of MCP-1 were reduced in the conditioned media of IPTD-MSCs treated with DMOG or DMOG plus TNF-α, but not with TNF-α alone. Levels of MMP-9, MMP-2, and IL-10 were not changed in response to various conditioning while L-1β was undetectable.

### Conditioned medium from IPTD-MSCs stimulated with DMOG promotes angiogenic activity of endothelial cells

**Fig. 9:**
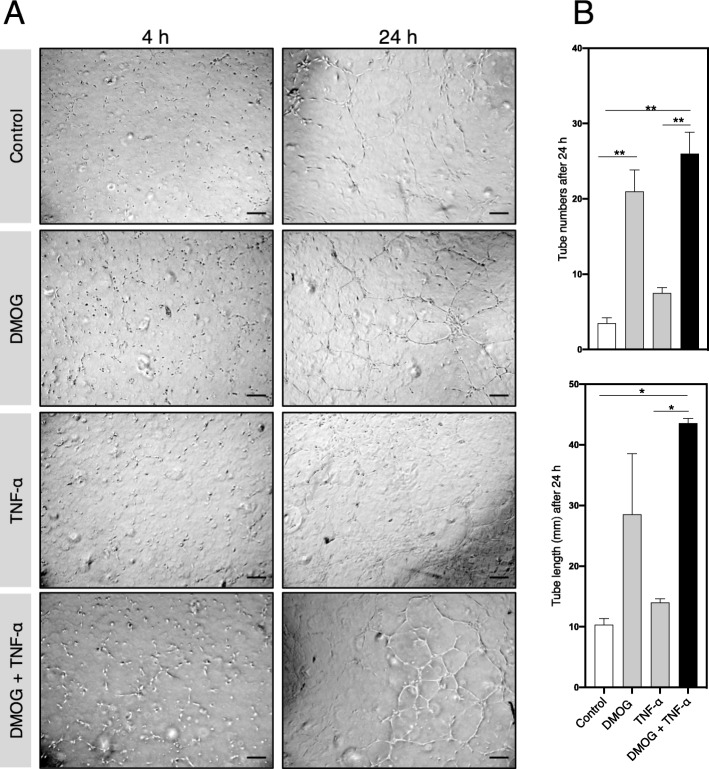
IPTD-MSC-derived media stimulates endothelial tube formation. **a** Representative photographs of endothelial cells cultured and treated with medium from conditioned and control IPTD-MSCs at 4 and 24 h post-culture. Scale bar, 500 μm. **b** Quantification is presented as total tube number and total tube length after 24 h. Duplicate samples were performed. Representative microscopic images are presented

Endothelial cell tube formation is an acknowledged angiogenic metric indicative of cell migration, adhesion, and re-organization. To test this, HUVECs were exposed to conditioned media from IPTD-MSCs stimulated with TNF-α, DMOG, or both. Four hours post-plating, HUVECs treated with various IPTD-MSC conditioned media displayed similar morphology without significant difference in tube number or length, regardless of the source of the media. By 24 h, endothelial tube formation was apparent (Fig. 9a). HUVECs incubated with media from IPTD-MSCs treated with DMOG (21.0 ± 2.0, *p* < 0.01) or DMOG + TNF-α (26.0 ± 2.0, p < 0.01) displayed increased numbers of cell tubes, compared to control (3.5 ± 0.5) (Fig. 9b). Similarly, tube length was significantly higher in HUVECs exposed to IPTD-MSC conditioned media stimulated with DMOG (28.6 ± 9.9 mm, *p* < 0.05) or DMOG + TNF-α (43.6 ± 0.8 mm, p < 0.05), compared to control (10.3 ± 1.0 mm) (Fig. 9b). TNF-α by itself had no effect on tube number or length, and TNF-α did not enhance the effects of DMOG (Fig. 9b; comparing the 2nd and the 4th bars), suggesting that DMOG is the sole stimulant to enhance IPTD-MSC-mediated angiogenesis in vitro.

## Discussion

We identified a MSC population that resides within pancreatic tissues, which can be separated during islet isolation. We named these cells intra-pancreatic tissue-derived (IPTD)-MSCs, in agreement with the recent call for nomenclature of MSCs in relation to their tissue of origin [37–39]. In culture, IPTD-MSCs displayed features similar to classic bone marrow- or umbilical cord blood-derived MSCs, including adherence to culture-grade plastic surfaces, spindle-shaped morphology, expression of appropriate surface markers (positive for CD90, CD105, and CD73, and negative for CD45, CD34, CD14 and HLA-DR), and capacity for proliferation and multilineage differentiation. Furthermore, when IPTD-MSCs were treated with a combination of TNF-α and DMOG, we observed (1) increased mRNA levels of *TSG-6*, *NRF2*, and *VEGF*; (2) increased secretion from IPTD-MSCs of VEGF, IL-6, IL-8, and IL-4; (3) decreased secretion of MCP-1; and (4) enhanced endothelial cell tube formation. Together, these results suggest IPTD-MSCs conditioned by TNF-α and DMOG have anti-inflammatory and pro-angiogenic potential.

The cell population, isolation, and culture method of IPTD-MSCs we described herein have both differences and similarities over other previously published MSCs [40]. In this study, cells were isolated from intra-pancreatic tissue as a part of islet isolation procedure from a single donor. IPTD-MSCs were harvested from an otherwise discarded component after routine pancreatic digestion and islet isolation. The GMP-compatible protocol used for culturing these cells led to the production of large numbers of highly purified MSCs. We deliberately selected CMRL-1066 as the base medium to propagate IPTD-MSCs because CMRL-1066 is routinely used to culture islets for transplantation, thus reducing the burden for future clinical translation. Additionally, we eliminated animal products in culture media by using hPL, which will lower the risks of infection, allergic reactions, and product variability. Similar to MSCs derived from other tissue sources, IPTD-MSCs are capable of differentiation into adipocyte, chondrocyte, and osteoblast lineages, demonstrating the multi-lineage potential of IPTD-MSCs.

This study demonstrates an approach that allows for harvesting islets and IPTD-MSCs simultaneously from a single donor under GMP conditions, facilitating direct clinical application. Harvesting IPTD-MSCs during human islet isolation makes the quality evaluation of isolated cells rapid and reliable and suggests opportunities for immediate clinical applications. Previously, autologous bone marrow-derived MSCs have been used simultaneously in living-related kidney transplant recipients [41]. Moreover, we expanded bone marrow-derived MSCs in the same medium of CMRL-1066 supplemented with hPL and found that these MSCs were similar in phenotype and characteristics compared to IPTD-MSCs, suggesting that our medium could be used to isolate MSCs from other tissue sources. IPTD-MSC culture medium used in this study is xenoprotein-free and cGMP-compatible. The isolated IPTD-MSCs were expandable and can be produced in large scale using this culture medium. Conventionally, fetal bovine serum is supplemented in selected culture media to promote the growth of MSCs from different tissue sources [42]. However, the use of non-human serum to culture cells carries the potential of transmitting infectious agents [43], immunizing effects [44], and lot-to-lot variability. In this regard, human platelet lysate has been used to replace fetal bovine serum for clinical-scale MSC expansion [45]. In these studies, hPL was supplemented in minimal essential medium (MEM) to culture MSCs. In the current study, we used hPL to supplement the CMRL-1066 that has been optimized for human islets culture, and the culture system employed herein allows for optimum survival of IPTD-MSCs. This is important since a single medium system can be used for both cell sources to facilitate co-transplantation of islets and IPTD-MSCs in future studies.

This study also highlights the benefit of harvesting multiple cell types from tissue fractions of a single donor organ as part of the islet isolation procedure. It is conceivable that immunophenotypic characterization and identification of additional novel cell types residing within this tissue fraction would be valuable to study pancreatic pathophysiology arising from various diseases.

MSCs are known to reduce inflammation and enhance healing, and these functions can be further manipulated ex vivo to enhance capacities for cell therapies. Compared to control, we found that IPTD-MSCs exposed to a combination of TNF-α and DMOG, compared to single reagents, exerted a better overall outcome. Except for *TSG-6* expression, no other molecules, including the secreted factors examined in this study, were affected by TNF-α treatment alone. In contrast, DMOG alone was able to induce *NRF2*, *VEGF* expression, as well as the secretion of VEGF, IL-6, IL-8, and IL-4. These results demonstrate a dominant effect of DMOG over TNF-α. However, TNF-α was able to augment the effects brought by DMOG in increasing the expression of *TSG-6*, *NRF2*, and *VEGF* and enhancing secretion from IPTD-MSCs of IL-6, IL-8, and IL-4. Regardless, the combination of TNF-α and DMOG appeared to be optimal for the examined outcomes, including the expression of *TSG-6*, *NRF2*, and *VEGF*; secretion of VEGF, IL-6, IL-8, and IL-4; and endothelial tube formation. To the best of our knowledge, this is the first study to show a beneficial effect on MSCs by conditioning with the combination of TNF-α and DMOG.

IL-4 levels were significantly increased by the combination of DMOG and TNF-α as compared to the control or DMOG alone, whereas IL-10 production was unchanged. This is in line with previous reports demonstrating that MSCs do not secrete IL-10, but stimulate other immune cells to secrete this cytokine [46]. MCP-1 (monocyte chemoattractant protein-1) is often increased upon treatment with inflammatory cytokines. We found that treatment of IPTD-MSCs with DMOG and TNF-α led to a reduction of MCP-1. Taken together, our results show the production of anti-inflammatory molecules in IPTD-MSCs. It remains to be determined if these human cells will provide protection in inflammatory settings.

VEGF, IL-6, IL-8, and MMP-9 are known pro-angiogenic factors [47–51], which may be responsible for the observed enhancement of endothelial cell tube formation. Upregulation and secretion of pro-angiogenic factors are important for several reasons: (i) MSCs from individuals with diabetes showed lowered angiogenic capacity [52] than those from individuals without diabetes, although another study reported that MSCs isolated from the bone marrow of T1D donors were phenotypically and functionally similar to those isolated from healthy individuals [53]; (ii) treatment of islets with the iron chelator deferoxamine stabilized HIF-α and enhanced islet VEGF levels [54]; and (iii) treatment with exogenous VEGF improves islet engraftment [55] and β-cell mass [56], in part through increased angiogenesis. Islet survival and function post-transplantation are adversely impacted by hypoxia [57]. Thus, processes that render islets hypoxia-resistant, such as increasing VEGF expression and secretion, should have beneficial effects in islet transplantation. The fact that the combination of DMOG and TNF-α also enhances *TSG-6*, *NRF2*, and *VEGF* gene expression from bone marrow-derived MSCs highlights the potential use of DMOG and TNF-α to condition MSCs other than IPTD-MSCs. Further, the upregulation of *TSG-6* in both IPTD-MSCs and bone marrow-derived MSCs underscores the concept of employing TSG-6 as a marker of anti-inflammatory capacity [58].

An interesting finding was the proximity of MSCs to islets within the pancreatic tissue, implying a possible role for these MSCs in protecting islets from metabolic stress and inflammation. Besides the potential anti-inflammatory and pro-angiogenic effects of IPTD-MSCs, future studies will explore whether extracellular vesicles (EV) [59] secreted by these cells are more effective at limiting autoimmune diseases such as T1D and uveoretinitis [60].

## Conclusion

In summary, the simultaneous isolation of human islets and intra-pancreatic tissue-derived MSCs was demonstrated. These IPTD-MSCs can be expanded in a clinically applicable culture system and potentiated ex vivo in their anti-inflammatory and pro-angiogenic properties. Such IPTD-MSCs, together with the islets originating from the same donor organ, may enhance islet transplantation outcome and other potential clinical applications.

## Data Availability

Please contact author for data requests.

## Abbreviations

DMOG: Dimethyloxallyl glycine
hPL: Human platelet lysate
HUVECs: Human umbilical vein endothelial cells
IL-1β: Interleukin 1β
IL-4: Interleukin 4
IL-6: Interleukin 6
IL-8: Interleukin 8
IL-10: Interleukin 10
IPTD: Intra-pancreatic tissue-derived
MCP-1: Monocyte chemoattractant protein-1
MMP-2: Matrix metallopeptidase 2
MMP-9: Matrix metallopeptidase 9
MSC: Mesenchymal stromal cell
NRF2: Nuclear factor erythroid 2–related factor 2
T1D: Type 1 diabetes
TSG-6: Tumor necrosis factor alpha-stimulated gene-6
VEGF: Vascular endothelial growth factor
